# Adaptation of Sensorimotor Coupling in Postural Control Is Impaired by Sleep Deprivation

**DOI:** 10.1371/journal.pone.0122340

**Published:** 2015-03-23

**Authors:** Stefane A. Aguiar, José A. Barela

**Affiliations:** 1 Institute of Physical Activity and Sport Sciences, Cruzeiro do Sul University, São Paulo, Brazil; 2 Institute of Biosciences, São Paulo State University, Rio Claro, Brazil; Tokai University, JAPAN

## Abstract

The purpose of the study was to investigate the effects of sleep deprivation (SD) in adaptation of the coupling between visual information and body sway in young adults’ postural control due to changes in optic flow characteristics. Fifteen young adults were kept awake for approximately 25 hours and formed the SD group, while fifteen adults who slept normally the night before the experiment participated as part of the control group. All participants stood as still as possible in a moving room before and after being exposed to one trial with higher amplitude and velocity of room movement. Postural performance and the coupling between visual information, provided by a moving room, and body sway were examined. Results showed that after an abrupt change in visual cues, larger amplitude, and higher velocity of the room, the influence of room motion on body sway was decreased in both groups. However, such a decrease was less pronounced in sleep deprived as compared to control subjects. Sleep deprived adults were able to adapt motor responses to the environmental change provided by the increase in room motion amplitude. Nevertheless, they were not as efficient as control subjects in doing so, which demonstrates that SD impairs the ability to adapt sensorimotor coupling while controlling posture when a perturbation occurs.

## Introduction

Although researchers and the general population agree that sleep is fundamental for quality of life, an increasing number of people experiences insufficient sleep regularly [1,2]. This scenario raises concerns due to the known deleterious effects of sleep deprivation (SD) on health and on performance on a number of different tasks usually executed while one is sleep deprived [3,4]. For this reason, several studies have investigated detrimental effects of SD on motor tasks in an attempt to determine risks of traffic and/or work-related accidents. For instance, performance on driving simulators [5], hand dexterity tasks [6], reaction time tests [7], and tasks involving hand-eye coordination [8] has been shown to deteriorate following SD.

One motor task that has received attention from researchers is the control of posture. Studies have demonstrated that postural control is clearly impaired by SD, which is evidenced through increased body sway [9–12]. These findings have even led to the suggestion that posturographic tests could be a good measure to detect sleepiness and, therefore, be used to identify people at riskand prevent accidents [13,14]. Even though negative effects of SD on postural control performance have been widely demonstrated, we are just beginning to understand the mechanisms underlying such performance decrements. To control posture, one needs to obtain sensory information coming from three main sources—visual, vestibular, and somatosensory channels—integrate these different cues and use them as a basis to generate appropriate muscle activity in order to achieve and maintain postural equilibrium and orientation [15]. As for the reasons why the functioning of such a system would be impaired due to SD, several researchers have claimed that difficulties with sensory integration could be responsible for the observed increased sway [9,11,16].

Aguiar and Barela [17] suggested that besides such difficulties, impairments in how sensory information is acquired and utilized as a basis to produce appropriate motor activity could be the reason why the control of posture is negatively affected by SD. The strategy used by the authors to examine such a relationship between sensory information and motor action was the moving room paradigm, in which optic flow characteristics are manipulated through the constant movement of a room in which participants are standing, and motor responses are recorded as correspondent changes in body sway resulting from room movement. Results showed that sleep deprived individuals demonstrated more variable and less coherent relationship between visual informationand body sway while controlling posture. The authors argued that these results confirm the hypothesis that SD alters the relationship between sensory information and motor action in the postural control system by demonstrating that sleep deprived subjects had difficulties uncoupling to irrelevant sensory information, which is an indication of a noisier postural control system [17].

Such results provide evidence that after SD individuals are less efficient in selecting the most relevant sensory information to be used as a basis for the production of muscle activity in order to control balance. Importantly, this was demonstrated under normal, unperturbed conditions, while subjects were simply standing upright as still as possible. Daily activities performed by sleep deprived individuals, however, also include tasks in which one has to deal with unexpected perturbations. For instance, among the occupations that cause most concerns regarding risks of accidents are drivers and doctors [18,19], professionals who have to handle different demands imposed unexpectedly to the task at hand—e.g., an obstacle that appears in the road requiring maneuvers from the driver or an unexpected development during surgery obligating the doctor to adopt a different procedure using a distinct set of motor skills.

Similarly, control of posture in common daily life situations involves the ability to deal with unexpected perturbations, which are also present while one is sleep-deprived. Evidence was provided to demonstrate that after SD individuals are less efficient at utilizing sensory information for the production of appropriate motor activity while controlling posture in unperturbed upright stance [17]. Nevertheless, no study has examined the capability of sleep-deprived subjects to adapt the relationship between sensory information and motor action in the postural control system after unexpected perturbations. Therefore, the purpose of this study was to investigate the effects of SD in the adaptation of the coupling between visual information and body sway in young adults’ postural control due to changes in optic flow characteristics.

## Materials and Methods

### Participants

Fifteen young adults were part of the SD group (23.60 ±4.5 years-old, 71.99 ±16.3 kg weight and 1.74 ±0.7 m height) and 15 young adults were part of the control group (27.35 ±5.5 years-old, 69.51 ±10.6 kg heavy and 1.72 ±0.6 m height). Subjects reported no diagnosed sleep disorders and none of them took any medication prior to or during the experiment. All participants had no musculoskeletal impairments that could interfere in the postural control task and had normal or corrected-to-normal vision, although no specific measures on visual accuracy other than regular medical evaluation were used.

### Ethics Statement

All participants gave their informed written consent prior to participation according to procedures approved by Cruzeiro do Sul University Review Ethics Committee (protocol number: CE/UCS-024/2013).

### Procedures

All participants were instructed to maintain regular sleep schedules three days before the experiment, which was monitored by sleep diaries. Information on hours of sleep in the night before the experiment for participants in the control group and hours of sleep in the night before the SD period for the SD group was obtained through these sleep diaries. A Portuguese version of the Pittsburgh Sleep Quality Index was administered in order to check the quality of sleep of participants in both groups [20].

In the day in which the experiment begun, participants in the SD group were instructed to wake up at their regular time, perform their normal activities during the day and arrive at the laboratory at 8 p.m., where they would remain awake until the morning of the next day to perform postural tasks. Subjects were monitored by experimenters during the whole night of SD and any type of physical exercise other than normal daily activities such as walking short distances was prohibited. Alcohol and caffeine were not allowed and food was provided (e.g., deli sandwich, cookies, juice, and water) every three hours during the night. During this SD night, participants performed activities such as chatting, playing cards, video games and reading. Participants in the control group were instructed to sleep normally the night before the experiment, not to drink alcohol and caffeine 24 hours prior to the experiment and to arrive at the laboratory in the morning to perform postural tasks. Postural control tasks were performed in the morning, between 8 and 11 a.m., for both groups. Since it has been demonstrated that performance in postural tasks fluctuates according to time of day [9], we expect restricting those tests to only the morning period and only in a small time window (3 hours) would strongly minimize any possible effect of time of day confounding with sleep deprivation effects and therefore biasing the results.

Participants stood upright inside of a moving room, which consisted of three walls and a roof (2.1 m long, 2.1m wide and 2.1 m height), mounted on wheels so that it could be moved back and forth by a servomotor mechanism while the floor remained stationary. The walls of the room were white with black stripes, creating a pattern of 42 wide vertical white and 22 cm wide vertical black stripes. Two 20-watt fluorescent lamps were attached to the ceiling in order to maintain consistent light throughout data collection.

One OPTOTRAK IRED marker (Certus—Northern Digital Inc.) was placed on the participant’s back (at the 8^th^ thoracic vertebra level) and another one was placed on the frontal wall of the moving room. These markers collected data from participant’s trunk sway and the moving room’s displacement, respectively, in the anterior-posterior, medial-lateral and vertical directions, with a sampling rate of 100 Hz. Although studies with postural control traditionally use force platforms and calculate center of pressure to represent overall body sway, experiments with the moving room paradigm are commonly performed using one marker on participants’ back with a position tracking system to measure body oscillation. This ensures both measures, room movement and participants’ body sway, are recorded using the same parameter (i.e. position in space) and has been normally accepted as a measure of overall body sway [21–23].

**Fig 1:**
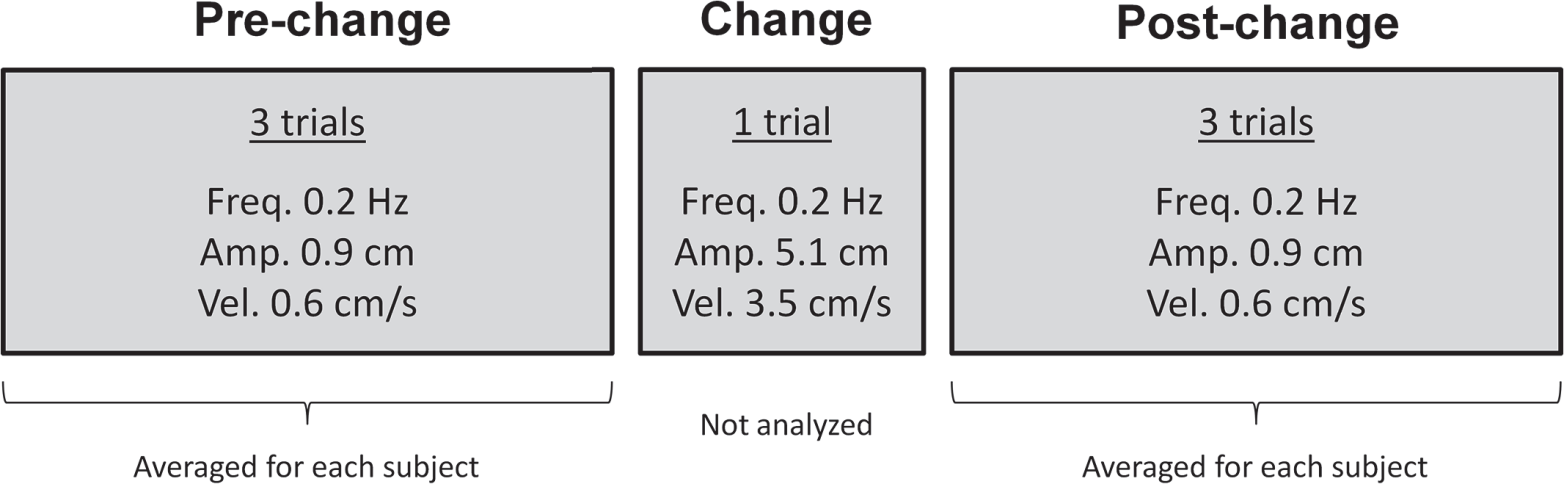
Schematic representation of the experimental design employed in this study. Number of trials and room movement parameters are described for pre-change, change, and post-change trials. Results for all dependent variables were averaged for each subject in the three trials of both pre- and post-change trials. Results of change trials were not included the in analyses.

In the postural control task all participants were asked to stand upright inside the moving room, as still as possible, looking at a target attached to the frontal wall of the room, 1 m distant. Each trial lasted 60 s and a total of seven trials were performed. In the first three trials, named *pre-change*, the room was moved with peak velocity of 0.6 cm/s, peak-to-peak amplitude of 0.9 cm and frequency of 0.2 Hz. In the fourth trial, named *change*, the room was moved with higher peak-to-peak amplitude, 5.1 cm, and peak velocity, 3.5 cm/s. In the last three trials, named *post-change*, the room was moved again with the same parameters used in the pre-change trials. Fig. 1 depicts a schematic representation of the experimental design employed in this study.

### Data Analysis

Analyses, including body sway magnitude and the relationship between body sway and room movement, were performed only in the anterior-posterior direction since this was the direction of the visual stimulus. The variable mean sway amplitude represented body sway magnitude and was calculated by obtaining the standard deviation of trunk position after the average trunk position and a first order polynomial were subtracted from the trunk position time series.

The relationship between body sway and visual stimulus was investigated using the variables gain, phase, and position and velocity variability. The variables gain and phase were obtained using a frequency response function (FRF). The FRF was calculated dividing the Fourier Transforms of trunk sway by the Fourier Transforms of room movement. Gain represented the absolute value, and phase the argument of this transfer function, calculated at the driving frequency (0.2 Hz). This analysis was employed in 5-second intervals throughout the trial (cycle-by-cycle FRF), generating 12 gain and phase values, which were averaged for each trial. Gain measured the magnitude of the influence of room movement on body sway. Gain values higher/lower than one indicate that the spectrum of body oscillation was higher/lower than the spectrum of room movement at the driving frequency. Phase represents the temporal relationship between body sway and room movement. Positive/negative phase values indicate that body sway was ahead/behind room movement.

Position and velocity variability were calculated after the signal from the position/velocity of the room movement was subtracted from the body sway time-series signal, generating a residual position/velocity trajectory. Position and velocity variability were calculated as the standard deviation of the position and velocity residual trajectories, respectively. Position and velocity variability represent body sway magnitude and velocity that occurred in frequencies different from the driving frequency.

### Statistical Analysis

After testing and fulfilling the normality and homogeneity of variance assumptions, parametric analyses were performed. A One-way analysis of variance (ANOVA) was conducted to evaluate the effect of group (SD and control) on the dependent variable hours of sleep in the night before the experiment. In order to check for possible differences in general sleep quality among participants in both groups, another One-way ANOVA was conducted to evaluate the effects of group (SD and control) on the dependent variable total score on the Pittsburgh Sleep Quality Index.

A 2 x 2 (Group x Trial) ANOVA with repeated measures on the last factor was conducted to evaluate the effects of group (SD and control) and trial (pre-change and post-change) on the dependent variable mean sway amplitude. Following, two 2 x 2 (Group x Trial) multivariate analyses of variance (MANOVAs) with repeated measures on the last factor were conducted to evaluate the effects of group (SD and control) and trial (pre-change and post-change) on the dependent variables gain and phase (first MANOVA) and position and velocity variability (second MANOVA). For all variables, the average from the first three trials was used for the pre-change estimate, and the average from the last three trials for the post-change estimate. When necessary, univariate analysis and Tukey HSD post hoc tests were utilized to test the main effects and interactions. The α-level for all analysis was set at 0.05.

## Results

ANOVA tests revealed that SD and control groups were not different in body mass, F(1, 28) = 0.24, p>0.05, η^2^ = 0.009, height, F(1, 28) = 0.30, p>0.05,η^2^ = 0.011, or age, F(1, 28) = 3.99, p = 0.056, η^2^ = 0.125, although this last factor showed a tendency for subjects in the control group to be older than individuals in the SD group. Sleep diaries showed that subjects in the SD group slept 7.24 (±2.31) hours the night before the SD procedures and subjects in the control groups slept 7.07 (±1.82) hours the night before the experiment, which was not significantly different between groups, F(1, 28) = 0.05, p>0.05, η^2^ = 0.002. The Pittsburgh Sleep Quality Index questionnaire demonstrated that sleep quality was similar for both groups, F(1, 28) = 0.20, p>0.05, η^2^ = 0.007, with the SD group scoring on average 5.53 (±1.72) and the control group 5.93 (±2.91) points. According to sleep diaries subjects in the SD group had been awake on average for 25.48 (±1.40) hours and in the control group for 2.45 (±0.83) hours when they performed the postural control tasks.

**Fig 2:**
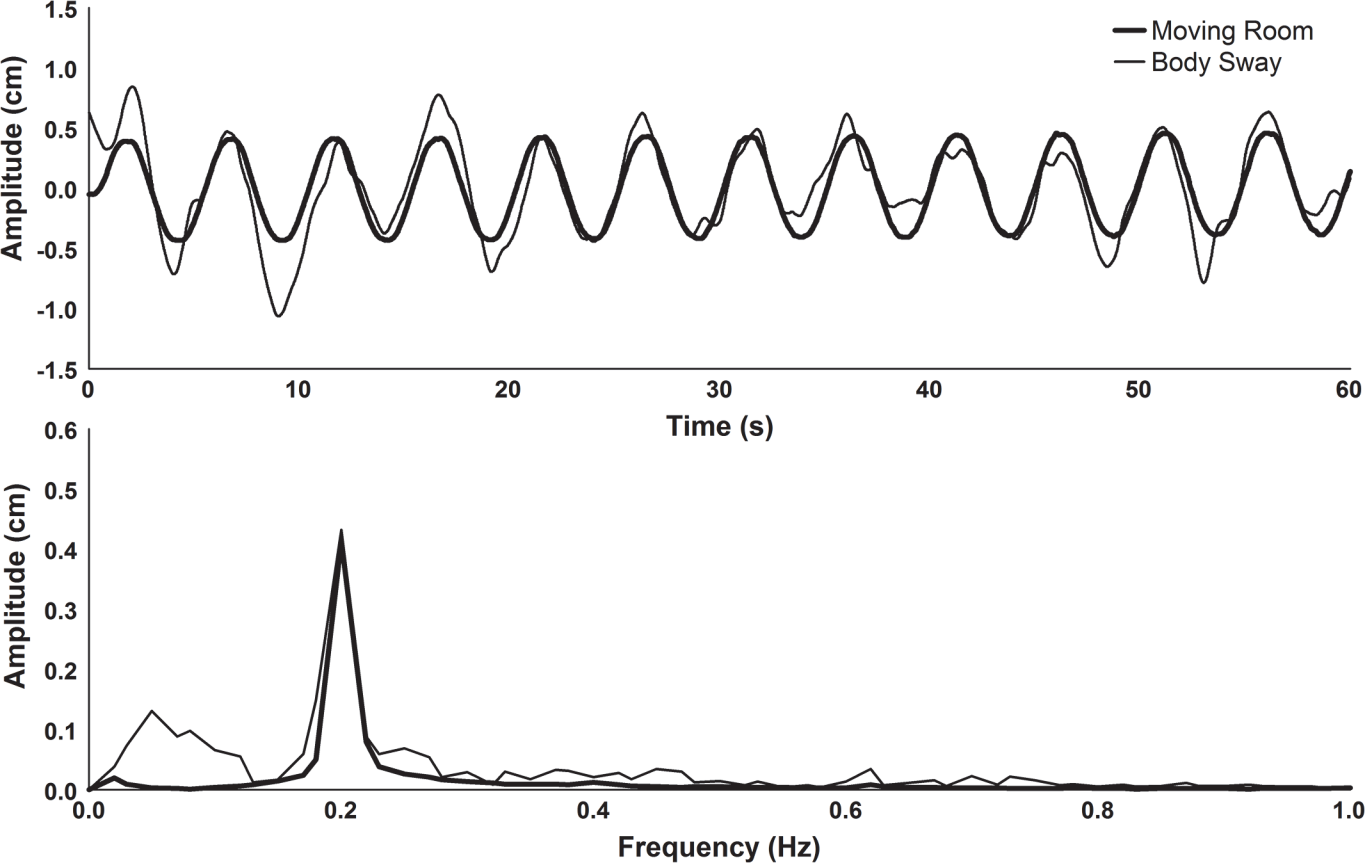
Time-series and amplitude spectra of body oscillation and moving room displacement of a representative subject of the control group during a pre-change trial.

Fig. 2 depicts time-series and amplitude spectra of body sway and moving room displacement from a representative subject of the control group. It can be observed that body sway accompanies room motion, moving back and forward, adopting the same oscillation frequency.

**Fig 3:**
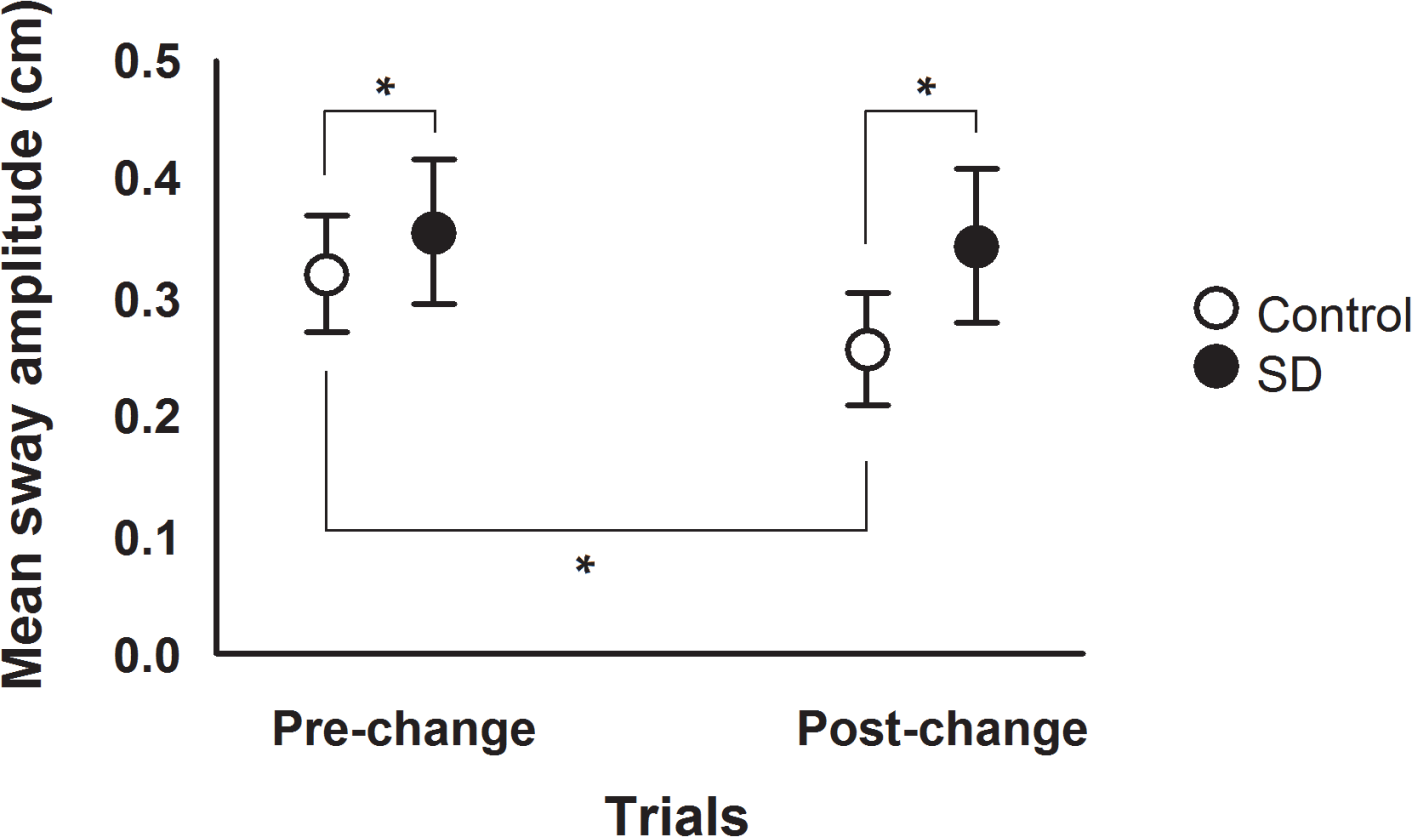
Mean and standard deviation values of mean sway amplitude in pre- and post-change trials for both sleep deprivation (SD) and control groups. Note: (*) represent significant pairwise comparisons, which was used for a group x trial interaction.

Overall body sway magnitude results are presented in Fig. 3, which depicts mean sway amplitude values in the pre- and post-change polled trials for both SD and control groups. ANOVA revealed group, F(1, 28) = 10.77, p<0.004, η^2^ = 0.278, and trial effect, F(1, 28) = 20.43, p<0.001, η^2^ = 0.422, and group and trial interaction, F(1, 28) = 9.90, p<0.005, η^2^ = 0.261. Post hoc tests on this interaction showed that mean sway amplitude decreased in the post-change compared to the pre-change trials only for the control group, and that mean sway amplitude was higher for the SD group compared to the control group in both pre-change and post-change trials.

**Fig 4:**
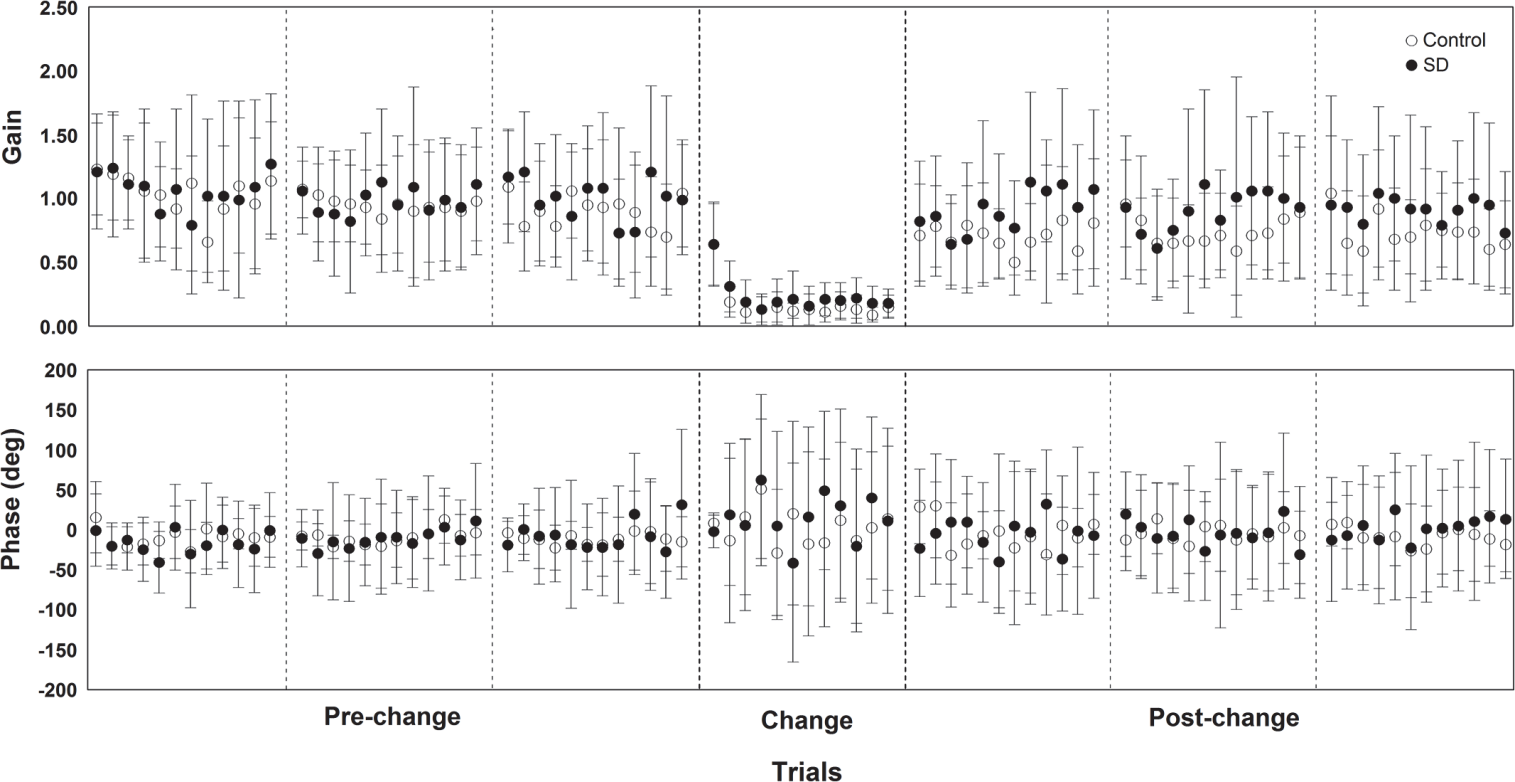
Mean and standard deviation values of gain and phase in all room movement cycles in pre-change, change, and post-change trials for both sleep deprivation (SD) and control groups.

**Fig 5:**
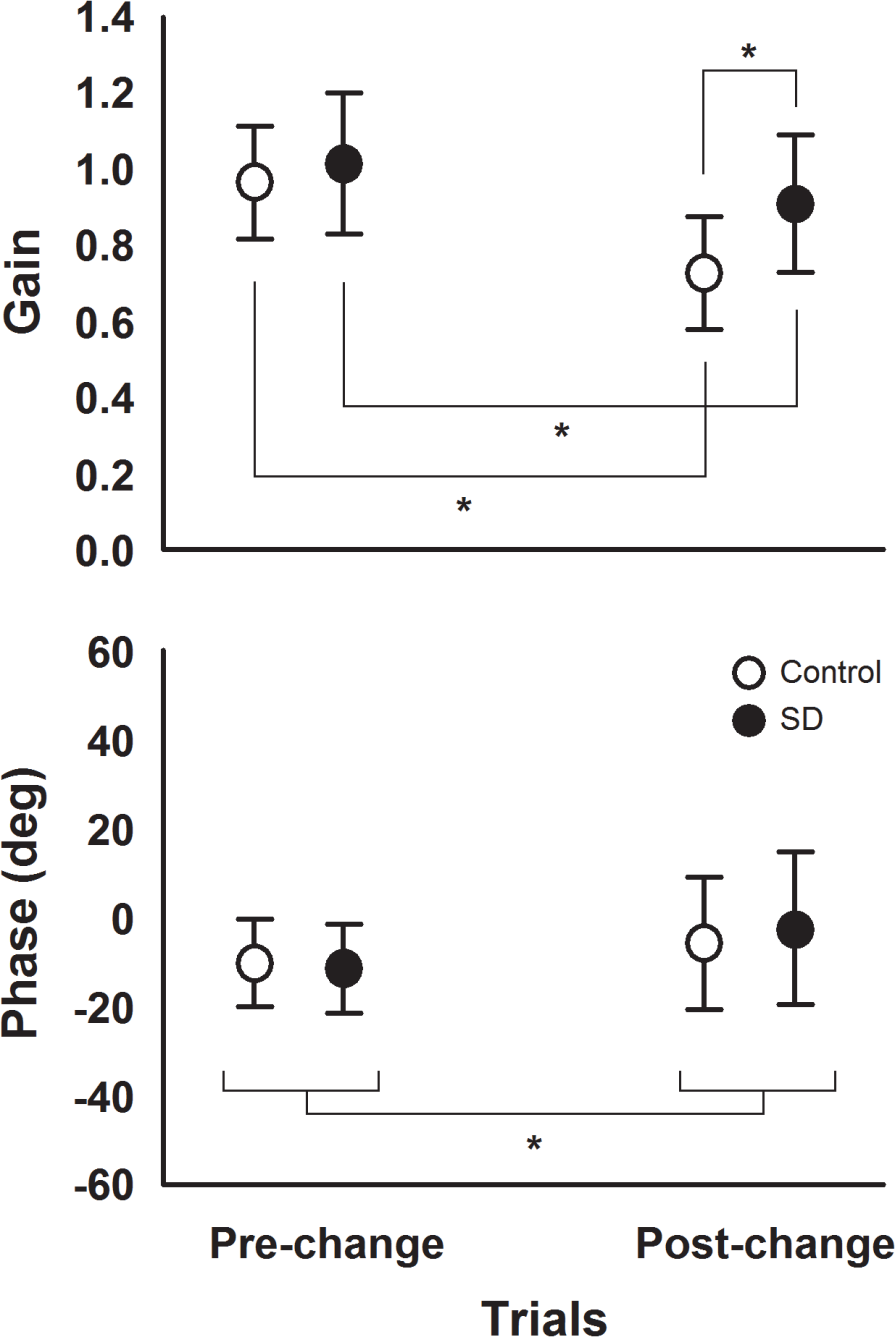
Mean and standard deviation values of gain and phase in pre- and post-change trials for both sleep deprivation (SD) and control groups. Note: (*) represent significant pairwise comparisons, which was used for group x trial interaction for gain and trial effect for phase.

Results on the magnitude of the influence of room movement on body sway and the temporal relationship between the two signals are presented in Fig. 4, which depicts gain and phase values during all cycles in pre-change, change, and post-change trials for both SD and control groups. Fig. 5 depicts gain and phase values in the pre- and post-change polled trials for both SD and control groups. MANOVA conducted with the variables gain and phase revealed trial effect, Wilk’s Lambda = 0.381, F(2, 27) = 21.92, p<0.001, η^2^ = 0.619, and group and trial interaction, Wilk’s Lambda = 0.787, F(2, 27) = 3.64, p<0.05, η^2^ = 0.213, but no group effect, Wilks’ Lambda = 0.850, F(2, 27) = 2.39, p>0.05, η^2^ = 0.150. Univariate analysis showed trial effect for gain, F(1, 28) = 43.27, p<0.001, η^2^ = 0.607, and phase, F(1, 28) = 5.56, p<0.03, η^2^ = 0.166, and group and trial interaction only for gain, F(1, 28) = 6.16, p<0.02, η^2^ = 0.180,. Phase values were higher in the post-change trials compared to the pre-change trials. The interaction group and trial showed that gain values decreased in the post-change compared to the pre-change trials for both SD and control groups and that gain values were higher for the SD compared to the control group in the post-change trials.

**Fig 6:**
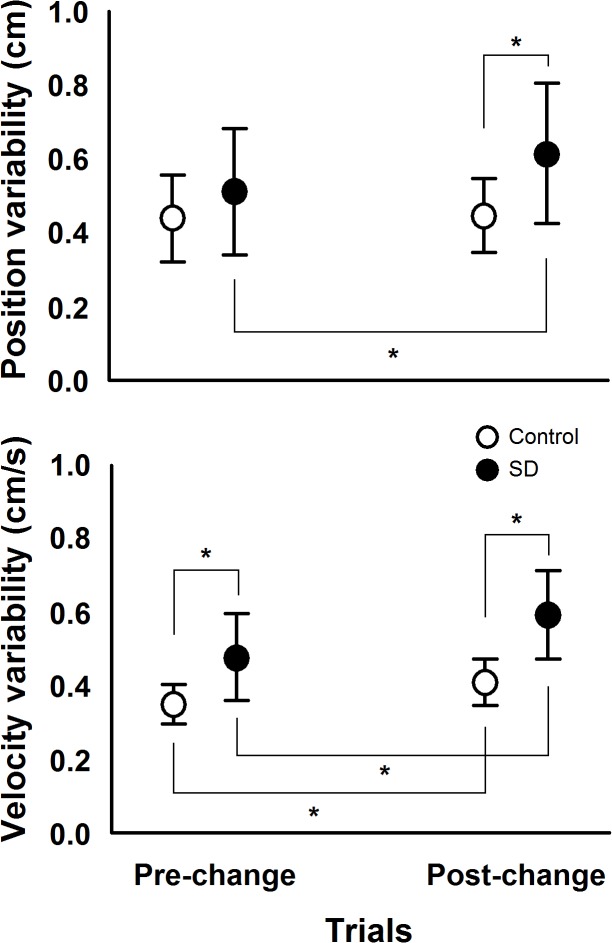
Mean and standard deviation values of position and velocity variability in pre- and post-change trials for both sleep deprivation (SD) and control groups. Note: (*) represent significant pairwise comparisons, which was used for group x trial interaction for both position and velocity variability.

Finally, results of body oscillation in frequencies other than the room movement frequency are shown in Fig. 6, which depicts position and velocity variability values in the pre- and post-change polled trials for both SD and control groups. MANOVA conducted with the variables position and velocity variability revealed group, Wilk’s Lambda = 0.556, F(2, 27) = 10.79, p<0.001, η^2^ = 0.444, and trial effect, Wilk’s Lambda = 0.276, F(2, 27) = 35.37, p<0.001, η^2^ = 0.724, and group and trial interaction, Wilk’s Lambda = 0.723, F(2, 27) = 5.18, p<0.02, η^2^ = 0.277.Univariate analysis revealed group effect for both position, F(1, 28) = 5.62, p<0.03, η^2^ = 0.167, and velocity variability, F(1, 28) = 22.38, p<0.001, η^2^ = 0.444, trial effect for both position, F(1, 28) = 7.11, p<0.02, η^2^ = 0.203, and velocity variability, F(1, 28) = 71.98, p<0.001, η^2^ = 0.720, and group and trial interaction for both position, F(1, 28) = 5.34, p<0.03, η^2^ = 0.160, and velocity variability, F(1, 28) = 7.28, p<0.02, η^2^ = 0.207,. Post hoc tests on group and trial interaction showed that position variability increased in the post-change trials compared to the pre-change trials only for the SD group and that position variability was higher in the SD compared to the control group in the post-change trials. Post hoc tests on group and trial interaction also revealed that velocity variability was higher for the SD compared to the control group in both pre- and post-change trials and that velocity variability increased in the post-change compared to the pre-change trials for both SD and control groups.

## Discussion

The aim of the present study was to investigate the effects of SD in the adaptation of the coupling between visual information and body sway in young adults’ postural control due to changes in optic flow characteristics. Our results showed that besides increasing overall body sway, SD also led to a less stable postural sway and visual information coupling, as evidenced by higher variability in sleep deprived individuals, especially after the change in room motion amplitude and velocity. Moreover, our results also showed that adaptation to changes in optic flow features was poorer after SD, which was clearly demonstrated by a smaller reduction of gain values in post-change trials observed for the SD (10.9%) compared to the control group individuals (25.0%).

Overall body sway magnitude was larger in sleep deprived compared to control subjects, which has been previously shown for both without any visual and with visual manipulation in the moving room condition [9,11,17,24]. Besides affecting the integration of sensory stimuli coming from different sources [9,11,16], it has been postulated that body sway increases after SD due to changes in the relationship between sensory information and motor activity required to control posture [17]. Our results also support such suggestion demonstrating that the coupling between body sway and visual information was more variable in sleep-deprived subjects, especially after changes in optic flow features.

One important result of the present study was that even sleep-deprived subjects were still strongly influenced by the visual manipulation provided by the movement of the room. Moreover, the observed coupling strength between body sway and room motion (gain values around 1) resembles the one observed for healthy young adults in normal rested conditions [17,25]. Therefore, based upon this result, we can suggest that although SD affects the use of sensory cues, it still cannot lead adults to ignore sensory cues which are available, even if it provides inaccurate information [25].

Our results clearly indicate that sensorimotor adaptation due to environmental changes was poorer in sleep-deprived individuals. Similarly to previous studies [25,26], after an abrupt increase of visual amplitude and velocity stimulus, participants show a reduction of body sway induction due to visual manipulation, observed in the gain values in the post-change trials. However, gain values reduction was smaller in sleep-deprived adults compared to the control peers, suggesting that such adaptation process had been affected by the lack of sleep. Such adaptive process due to abrupt changes in sensory cues has been suggested to reflect a sensory re-weighting process, which is based on the ability to up- or down-weight the influence of a given sensory source on postural control [27–31]. Once stimuli coming from the visual sensory channel are detected to be inaccurate, which occurs when the amplitude of room motion is too large as in the change trial used in this study, the estimate of body position, performed by the central nervous system, attributes less importance or weight to visual cues and attributes more importance tothe cues coming from the other sensory channels, resulting in a drop in gain values to the visual information [25,32].

The smaller reduction in gain in the SD group after the increase in room movement amplitude and velocity demonstrates that such process of sensory re-weighting is less efficient in adults after SD. Therefore, the ability to select the most relevant sensory cues to use as basis for the production of appropriate motor activity to control posture is impaired due to SD. Impairments in such ability were demonstrated by Aguiar and Barela [17] during unperturbed quiet stance, evidenced by lower precision and stability in the coupling between body sway and visual information, and our present results extendprevious findings by showing that when adaptation to external perturbations is required subjects are also less efficient in selecting the most relevant sensory cues after SD.

The fact that sleep deprived individuals did not adapt postural control performance to changes in optic flow characteristics as efficiently as their peers without SD was further evidenced by the decrease in mean sway amplitude after the change trial which was only observed in the control group. Therefore, it is evident that after the trial with larger amplitude and higher velocity of room motion sleep-deprived subjects not only reduced gain values with smaller magnitude compared to the control group and became less stable in relating body sway to visual information, but also did not adapt postural control by displaying overall body sway decrease, contrary to that observed for the control group. These results are in accordance with the suggestion that increased body sway in sleep deprived subjects could be intimately related to impairments in how sensory information is used related to the production of proper motor activity while controlling balance [17]. Aguiar and Barela have shown that larger body sway magnitude was observed simultaneously with impairments in sensorimotor coupling in sleep deprived subjects during quiet stance conditions [17], and we can add that after a need to adapt posture to environmental changes such result can also be observed. The diminished stability in sensorimotor coupling, shown by the higher values of position and velocity variability in sleep deprived subjects compared to the control group after the change trial provides evidence that following SD individuals displayed body sway in frequencies other than the visual stimulus frequency. In this way, sleep deprived subjects presented body oscillation in a wider range of frequencies, not only the driving frequency, which results in larger overall body sway as compared to the control group. Such behavior is referred to as a noisier postural control system, which besides coupling body sway to visual stimulus also displays coupling to other stimuli and, therefore, is said to present difficulties uncoupling to irrelevant sensory information [17].

The impairments in sensorimotor adaptation observed in our results have important functional consequences. Since it was demonstrated that the ability to adapt how sensory information is integrated and used to generate appropriate motor activity is compromised after SD, performance in a constantly changing environment in activities that require sensory-motor adaptation might be severely compromised when one is sleep deprived. Such activities include, for example, driving and operating machines, which require among other issues the constant attention of the driver towards other cars’ movements in order to respond appropriately as well as a worker’s ability to handle unexpected problems with equipment while performing a given task, respectively. Importantly, these activities have been shown to be performed frequently by individuals in conditions of SD [19]. Therefore, poor adaptation to environmental changes in sensorimotor tasks following SD represents a great concern regarding risks of traffic and/or work-related accidents.

Deficits caused by SD on visual attention [33] are in agreement with our findings and could be proximately related to them. In this case, the cognitive processing of visual information could also be affected by SD. The well documented detrimental effects of SD on cognitive functions [34] certainly could have contributed to the observed poor adaptation of sensorimotor coupling. For example, inappropriate inhibition of motor responses could play a role in those deficits [35,36], especially considering its known impairments following sleep deprivation [37]. Although the extent to which cognition participates in sensorimotor coupling in the moving room paradigm has been investigated recently [38], unfortunately, there is no evidence as to how this happens in sleep deprived individuals and future studies should address this issue.

A key limitation of this study is that effects of SD on performance were tested among different subjects, which increases variability in the data. Nevertheless, with the present experimental design it would not be possible to test the same individuals before and after SD because once subjects are exposed to the trial with larger amplitude and velocity of room movement, sensorimotor coupling is changed through a decrease in gain values, which could bias results if subjects were tested a second time. Another possible limitation of this study is the fact that age was slightly different among groups, with subjects in the SD group being slightly younger (23.60 ± 4.5 years-old) than in the control group (27.35 ± 5.5 years-old). Although this could be a limitation, the fact that the effect was only marginal (p = 0.056), togetherwith the lack of evidence for differences in postural performances among individuals in the age range of 20–25 compared to 25–30 strongly suggest that present results were not biased by group ages. In addition, Pearson correlation tests between age and each of the dependent variables were ran indicating weak or no correlation in every case (higher coefficient of correlation was *r* = 0.36), and ANCOVAs with age as covariate were also conducted for each dependent variable, revealing similar results as the ones reported and suggesting that age does not seem to affect the observed results.

In sum, our results demonstrate that SD affects postural control by increasing body sway, which could be related to impairments caused by SD on the ability to use sensory information accurately to produce appropriate motor activity while controlling posture. In addition, sleep-deprived individuals also show poor adaptation of such sensorimotor coupling while controlling posture when environmental changes occur, leading to serious consequences related to risk of accidents during the execution of sensorimotor tasks after SD.
